# Development of Arterial Thrombosis in a Patient on Therapeutic Anticoagulation for COVID-19

**DOI:** 10.7759/cureus.17644

**Published:** 2021-09-01

**Authors:** Essam K Nagori, Rory Smith, Fernando Sorto, Mufadda Hasan

**Affiliations:** 1 Internal Medicine, Arrowhead Regional Medical Center, Colton, USA; 2 Pulmonary and Critical Care Medicine, Arrowhead Regional Medical Center, Colton, USA

**Keywords:** sars-cov-2, hypercoagulable, arterial, thrombosis, covid-19, anticoagulation

## Abstract

The severe acute respiratory syndrome coronavirus-2 (SARS-CoV2) virus has been known to manifest various non-pulmonary complications in the affected patient, including both venous and less frequently, arterial thrombosis. Ongoing research is necessary to determine who might benefit from therapeutic anticoagulation as well as potentially develop an algorithm to predict thromboembolic events in this patient population. We present a case of a 65-year-old man with a past medical history of hypertension, diabetes type 2, and a previous cerebrovascular accident who was admitted with acute hypoxemic respiratory failure due to coronavirus disease 2019 (COVID-19). On admission to the hospital, the patient was initiated on therapeutic anticoagulation. Subsequently, he developed left lower limb ischemia. Imaging discovered arterial thrombosis in the bilateral deep femoral and left popliteal arteries. Consequently, the patient required catheter directed thrombolysis for partial reperfusion. Unfortunately, the patient succumbed to the complications of COVID-19. This case is notable in that it highlights the arterial thrombophilia associated with COVID-19 despite early intervention with therapeutic anticoagulation.

## Introduction

Prior to the spread of coronavirus disease 2019 (COVID-19), critically ill patients in the ICU have been predisposed to thrombotic events due to an array of contributing factors [1,2]. COVID-19 has not only caused respiratory complications but also affected multiple organ systems [3]. Furthermore, the COVID-19 pandemic has also caused physical distress in non-COVID-19 patients [4]. As the pandemic continues and an increasing number of patients are admitted to the ICU, it is crucial to highlight the escalating frequency of thromboembolic events. Several recent publications indicate a predilection for venous thromboembolic (VTE) events associated with COVID-19. However, there have been infrequent discussions regarding arterial thrombosis [5-9].

A French study of patients with severe COVID-19 infection indicated patients receiving prophylactic anticoagulation had a 100% VTE rate as compared to 56% in the therapeutic anticoagulation group [10]. This underscores the importance of early screening and therapeutic anticoagulation for high-risk patients, an intervention supported by the growing literature on COVID-19 and VTE [11].

## Case presentation

**Figure 1:**
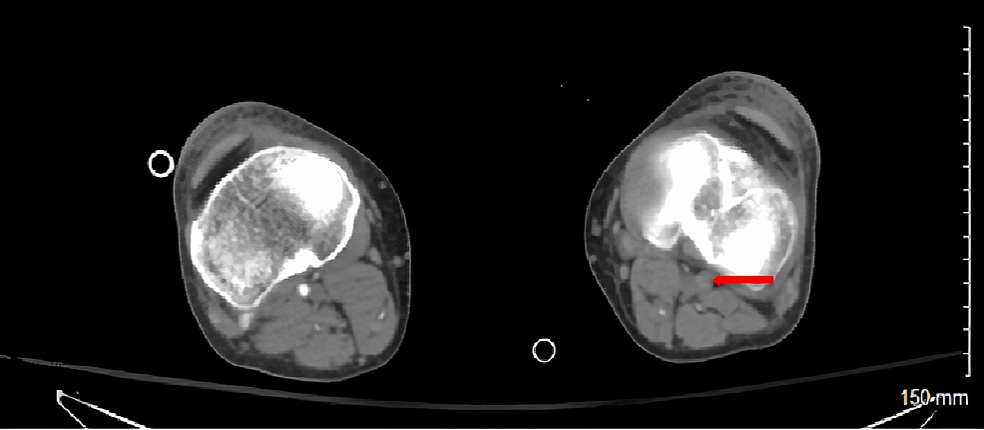
CT angiogram of lower extremities identifying left popliteal occlusion (red arrow).

Here we describe a case of a 65-year-old Caucasian man with a past medical history of diabetes type two, hypertension, and a previous cerebral vascular accident (CVA) who presented to the emergency department in December of 2020 with a chief complaint of shortness of breath. On admission, he tested positive for COVID-19. His blood tests were remarkable for a D-dimer elevated to 870 DDUng/ml. He had no complaints consistent with limb ischemia and his physical examination was unremarkable for signs of reduced peripheral tissue perfusion. Following the institution’s protocol, he was started on continuous heparin infusion while in the emergency department due to concerns for increased VTE risk. With the need for continued anticoagulation, he was transitioned to therapeutic dose enoxaparin on day two of hospitalization. The patient developed worsening respiratory failure and an inability to tolerate noninvasive mechanical ventilation (NIMV) and, therefore, was intubated on day five of hospitalization. Several hours after being placed on mechanical ventilation, the patient was noted to have an acute change to his left lower extremity with discoloration and diminished pulses. A documented assessment performed earlier in the day revealed the patient had +1 left dorsalis pedis (DP) pulse. The DP pulse had become absent and was undetectable via doppler examination. Additionally, the posterior tibial (PT) pulse on the left side was no longer palpable, however was faintly detected on Doppler. A computed tomography (CT) angiogram of the left lower extremity was obtained and therapeutic enoxaparin was reverted back to a continuous heparin drip in anticipation of any surgical interventions. The CT angiogram was notable for arterial thrombosis of the bilateral deep femoral and the left popliteal arteries, with probable one-vessel runoff in the left calf via the reconstituted left mid PT artery (Figure 1).

**Figure 2:**
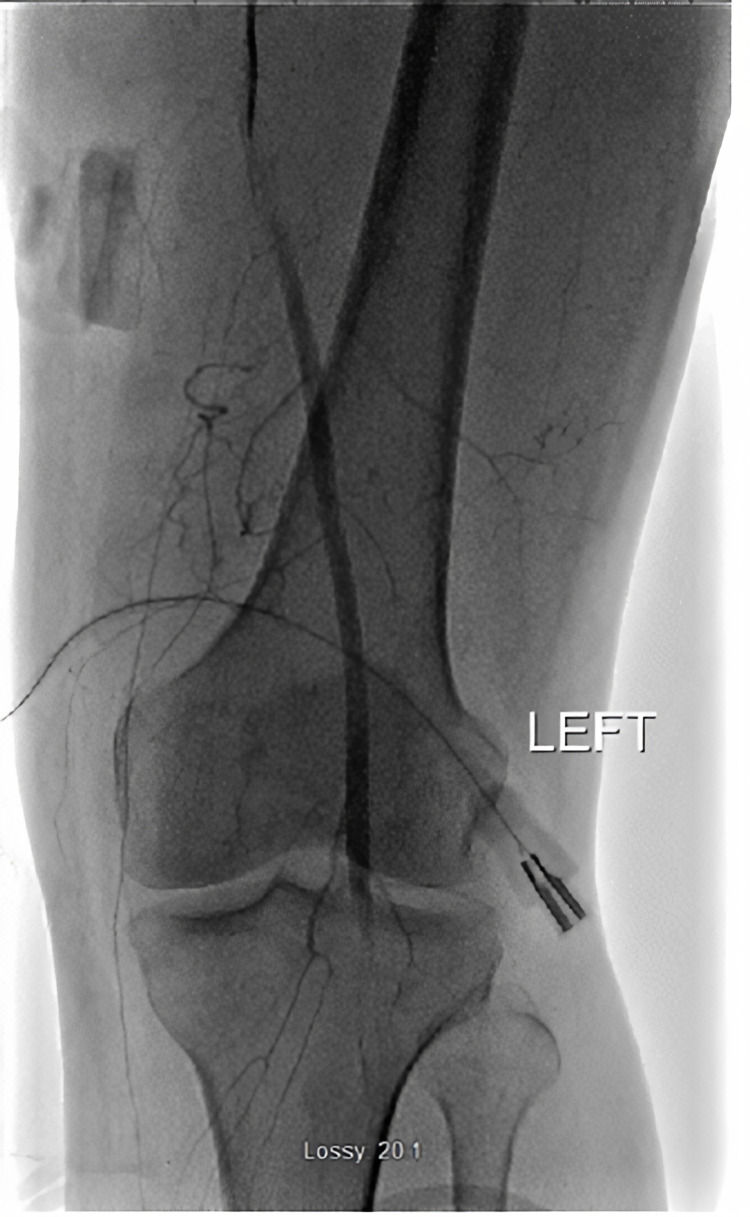
Catheter directed contrast evaluation of left popliteal artery occlusion prior to thrombolysis.

Also noted was diffuse atherosclerotic disease in bilateral superficial femoral arteries. Given the acute change and risk to limb, interventional radiology performed catheter directed thrombolysis with alteplase to the left tibioperoneal trunk. Figure 2 shows the vessel occlusion prior to thrombolytic infusion.

During the next few days, the left foot exhibited capillary refill of two seconds and a biphasic PT signal while the DP signal remained absent. Due to the concern for thromboembolic disease, a transthoracic echocardiogram without a bubble study was completed. The study was limited in overall visualization of the endocardium but was able to identify the left ventricular ejection fraction estimate to be 75% in normal sinus rhythm. Multiple electrocardiograms taken during the patient's hospitalization only indicated sinus tachycardia. A review of the electronic medical records indicated that he did not have any prior hypercoagulable workup completed, nor was one further investigated interim. The patient remained critically ill requiring vasopressors and high positive end-expiratory pressure (PEEP) on the ventilator and was therefore deemed too unstable for formal vascular surgery. On day 17 of admission, the patient was successfully extubated to high-flow nasal cannula and maintained on a heparin infusion until day 21 of hospitalization, at which time he was switched to full dose enoxaparin for anticoagulation. Unfortunately, the patient had an acute decompensation on day 21 and ultimately succumbed to his illness on day 25 of hospitalization.

## Discussion

The use of deep venous thrombosis (DVT) prophylaxis has been well established in critically ill patients [1]. In the midst of the COVID-19 pandemic, it has been difficult to establish guidelines for the appropriate administration of prophylactic versus therapeutic anticoagulation in this patient population. Prior studies have shown that in addition to being at risk for venous thrombosis, patients suffering from COVID-19 are also at risk for arterial thrombosis [5,6]. Furthermore, studies have also reported D-dimer as a prognostic marker of mortality in COVID-19 patients treated with tocilizumab [12]. This case highlights the significant coagulopathy associated with COVID-19 even while on therapeutic anticoagulation, as previously acknowledged by Llitjos et al. [11]. We identified a patient who developed an arterial thrombosis despite therapeutic anticoagulation and no known prior diagnosis of peripheral arterial disease or arrhythmia such as atrial fibrillation. Currently, there is no predictive algorithm or validated criteria for identifying patients who may develop a thrombotic event.

A recent publication by Ferrari et al. [13] determined thrombophilic markers did not have a correlation to disease severity. Additionally, histopathologic studies have indicated evidence of thrombosis in segmental and sub-segmental pulmonary arterial vessels despite the use of prophylactic anticoagulation [14]. Combining the knowledge from these two studies indicate a need for vigilance of clinical status prior to and during anticoagulant use. Currently, the consensus as reported by CHEST indicates that patients who are acutely ill or critically ill with COVID-19 should receive prophylactic anticoagulation over no prophylaxis. Low molecular-weight heparin (LMWH) or fondaparinux should be used over unfractionated heparin (UFH). These are to be used before direct oral anticoagulants (DOACs). CHEST has also recommended against full dose anticoagulation without evidence of thromboembolic disease. These anticoagulants should be used in accordance with existing guideline dosing [15].

## Conclusions

Given these early studies and case reports, it is imperative to approach each case individually and determine the risks and benefits of prophylactic versus therapeutic anticoagulation in patients suffering from COVID-19. Clinicians caring for patients with COVID-19 should consider maintaining a low threshold to increase anticoagulation from prophylactic dosing to therapeutic levels in the severely ill, particularly if bleeding risk is low and there are no contraindications to therapeutic anticoagulation. These decisions should be made while maintaining adherence to clinical guidelines if possible. Moreover, clinicians should remain vigilant in identifying the development of acute arterial thromboses in patients with severe COVID-19 infection even while receiving therapeutic anticoagulation. Further studies are needed to better understand the factors which predispose patients to thromboembolic disease and anticoagulation failure in the setting of a COVID-19 infection.

## References

[1] Lewis TC, Cortes J, Altshuler D, Papadopoulos J (2019). Venous thromboembolism prophylaxis: a narrative review with a focus on the high-risk critically ill patient. J Intensive Care Med.

[2] Previtali E, Bucciarelli P, Passamonti SM, Martinelli I (2011). Risk factors for venous and arterial thrombosis. Blood Transfus.

[3] Sharma K, Desai HD, Patoliya JV, Jadeja DM, Gadhiya D (2021). Takotsubo syndrome a rare entity in COVID-19: a systemic review-focus on biomarkers, imaging, treatment, and outcome. SN Compr Clin Med.

[4] Desai HD, Sharma K, Jadeja DM, Desai HM, Moliya P (2020). COVID-19 pandemic induced stress cardiomyopathy: a literature review. Int J Cardiol Heart Vasc.

[5] Klok FA, Kruip MJ, van der Meer NJ (2020). Incidence of thrombotic complications in critically ill ICU patients with COVID-19. Thromb Res.

[6] Mestres G, Puigmacià R, Blanco C, Yugueros X, Esturrica M, Riambau V (2020). Risk of peripheral arterial thrombosis in COVID-19. J Vasc Surg.

[7] Ferguson K, Quail N, Kewin P, Blyth KG (2020). COVID-19 associated with extensive pulmonary arterial, intracardiac and peripheral arterial thrombosis. BMJ Case Rep.

[8] Kashi M, Jacquin A, Dakhil B, Zaimi R, Mahé E, Tella E, Bagan P (2020). Severe arterial thrombosis associated with Covid-19 infection. Thromb Res.

[9] Iba T, Levy JH, Connors JM, Warkentin TE, Thachil J, Levi M (2020). The unique characteristics of COVID-19 coagulopathy. Crit Care.

[10] Desai HD, Sharma K, Parikh A (2021). Predictors of mortality amongst tocilizumab administered COVID-19 Asian Indians: a predictive study from a tertiary care centre. Cureus.

[11] Llitjos JF, Leclerc M, Chochois C, Monsallier JM, Ramakers M, Auvray M, Merouani K (2020). High incidence of venous thromboembolic events in anticoagulated severe COVID-19 patients. J Thromb Haemost.

[12] Miesbach W, Makris M (2020). COVID-19: coagulopathy, risk of thrombosis, and the rationale for anticoagulation. Clin Appl Thromb Hemost.

[13] Ferrari E, Sartre B, Squara F (2020). High prevalence of acquired thrombophilia without prognosis value in patients with coronavirus disease 2019. J Am Heart Assoc.

[14] Lax SF, Skok K, Zechner P (2020). Pulmonary arterial thrombosis in COVID-19 with fatal outcome: results from a prospective, single-center, clinicopathologic case series. Ann Intern Med.

[15] Moores LK, Tritschler T, Brosnahan S (2020). Prevention, diagnosis, and treatment of VTE in patients with coronavirus disease 2019: CHEST guideline and expert panel report. Chest.

